# The use of syndromic surveillance for decision-making during the H1N1 pandemic: A qualitative study

**DOI:** 10.1186/1471-2458-12-929

**Published:** 2012-10-30

**Authors:** Anna Chu, Rachel Savage, Don Willison, Natasha S Crowcroft, Laura C Rosella, Doug Sider, Jason Garay, Ian Gemmill, Anne-Luise Winter, Richard F Davies, Ian Johnson

**Affiliations:** 1 Public Health Ontario, Toronto, ON, Canada; 2 Dalla Lana School of Public Health, University of Toronto, Toronto, ON, Canada; 3 Department of Clinical Epidemiology and Biostatistics, McMaster University, Hamilton, ON, Canada; 4 Department of Laboratory Medicine and Pathobiology, University of Toronto, Toronto, ON, Canada; 5 Kingston, Frontenac and Lennox and Addington Public Health, Kingston, ON, Canada; 6 University of Ottawa Heart Institute, Ottawa, ON, Canada

**Keywords:** Decision making, Pandemic influenza, Public health, Surveillance, Syndromic surveillance

## Abstract

**Background:**

Although an increasing number of studies are documenting uses of syndromic surveillance by front line public health, few detail the value added from linking syndromic data to public health decision-making. This study seeks to understand how syndromic data informed specific public health actions during the 2009 H1N1 pandemic.

**Methods:**

Semi-structured telephone interviews were conducted with participants from Ontario’s public health departments, the provincial ministry of health and federal public health agency to gather information about syndromic surveillance systems used and the role of syndromic data in informing specific public health actions taken during the pandemic. Responses were compared with how the same decisions were made by non-syndromic surveillance users.

**Results:**

Findings from 56 interviews (82% response) show that syndromic data were most used for monitoring virus activity, measuring impact on the health care system and informing the opening of influenza assessment centres in several jurisdictions, and supporting communications and messaging, rather than its intended purpose of early outbreak detection. Syndromic data had limited impact on decisions that involved the operation of immunization clinics, school closures, sending information letters home with school children or providing recommendations to health care providers. Both syndromic surveillance users and non-users reported that guidance from the provincial ministry of health, communications with stakeholders and vaccine availability were driving factors in these public health decisions.

**Conclusions:**

Syndromic surveillance had limited use in decision-making during the 2009 H1N1 pandemic in Ontario. This study provides insights into the reasons why this occurred. Despite this, syndromic data were valued for providing situational awareness and confidence to support public communications and recommendations. Developing an understanding of how syndromic data are utilized during public health events provides valuable evidence to support future investments in public health surveillance.

## Background

Syndromic surveillance, which uses pre-diagnostic data for public health surveillance purposes, was initially incorporated into public health surveillance for the early detection of infectious disease outbreaks to improve on the inherent time lags with surveillance data that are reliant upon clinical or laboratory confirmation. Accordingly, evaluations of syndromic surveillance tend to focus on measuring the sensitivity and specificity of systems for outbreak detection [1-4].

In 2004, the United States (US) Centers for Disease Control and Prevention (CDC) published a framework for evaluating public health surveillance systems for early detection of outbreaks (Table 1) [5]. However, some components have received less attention, particularly the measurement of usefulness in public health decision-making [6-8]. This may be due in part to difficulties in measurement. The CDC recommends assessing usefulness by determining the impact or value added from linking surveillance data to public health actions.

**Table 1:** US CDC framework for evaluating public health surveillance systems for early detection of outbreaks

**System description**	**Outbreak detection**	**System experience**
· Purpose	· Timeliness	· Usefulness
· Stakeholders	· Validity (including data quality)	· Flexibility
· Operation		· Acceptability
		· Portability
		· Stability
		· Cost

Although an increasing number of studies are documenting uses of syndromic surveillance by front line public health, few provide information on how syndromic surveillance is linked with public health action [9-13]. In US case studies of potentially significant public health events, syndromic surveillance was reported to be useful for monitoring health impact, but had limited impact on public health responses [9]. Similarly, interviews with US health departments showed that although syndromic surveillance was initially implemented for early outbreak detection, in practice, health departments used syndromic data for situational awareness, to confirm or rule out events of significance and to support traditional public health investigations and surveillance [14]. Additionally, health departments sought to verify fewer than 15% of alerts generated from the syndromic surveillance system because the alert was obviously a false positive or resource constraints limited the ability for follow-up [14]. Lastly, a web-survey of Ontario’s 36 local public health departments, the provincial ministry of health and federal public health agency found that syndromic data were considered less useful for informing public health decisions than laboratory testing data during the 2009 influenza pandemic (A(H1N1)pdm09) [15]. Nonetheless, 70% of organizations with access to emergency department (ED) screening data felt that these data were essential for informing public health decisions on how to respond locally to A(H1N1)pdm09.

We conducted a qualitative follow-up to the aforementioned Ontario survey to describe syndromic surveillance system experiences for systems used in Ontario during the 2009 influenza pandemic, including whether and how syndromic data informed specific public health actions. For comparison, we included a sample of public health departments without access to local syndromic data to describe what information informed their public health actions.

## Methods

### Setting

During the 2009 influenza pandemic in Ontario, influenza surveillance was comprised primarily of traditional laboratory based surveillance and case investigation, weekly reporting of sentinel physician influenza-like illness (ILI) consultations, over-the-counter respiratory drug sales and antiviral prescriptions, daily monitoring of calls to Telehealth (Ontario’s free, confidential telephone service that the public may call to get health advice from registered nurses), and local syndromic surveillance. The latter refers to locally developed and implemented systems designed to collect, analyze and use local data on school absenteeism, workplace absenteeism, sentinel physician ILI consultations and/or ED visits.

### Participant selection

Following completion of the 2010 web-survey [15], we emailed invitations to medical officers of health and epidemiologists from the 31/36 local public health departments who consented to being re-contacted, as well as the provincial ministry of health (public health and emergency management branches) and the federal public health agency (respiratory division) to share summary results and to request further participation in the evaluation. The organizations contacted comprised all those responsible for public health surveillance of A(H1N1)pdm09 at the local, provincial and federal level in Ontario. Their views, therefore, were felt to be representative of experiences in Ontario with operationalizing and using public health surveillance data (including syndromic data). Organizations interested in continuing (33/38) were asked to identify two participants involved in A(H1N1)pdm09 surveillance for a 60-minute semi-structured interview - one person who could answer questions on the decision-making process during the pandemic for their organization (e.g. medical officer of health or manager), and another who was familiar with the operation of surveillance systems used (e.g. epidemiologist).

### Data collection

Two interview guides, one for syndromic surveillance system users (SSU) and another for non-users (NSU), informed in part by the CDC framework, guided the semi-structured interviews [5]. SSUs were defined as organizations that systematically collect syndromic data in (near) real-time, perform rapid analysis on the data as it is received and disseminate findings to support response. Interview questions were reviewed by the study’s Advisory Committee and pilot tested with four participants from the field. Each guide was distributed to participants in advance for review.

The interview guide consisted of three sections [see Additional file 1]. In section 1, descriptive information on the syndromic surveillance system(s) (e.g. purpose, uses, data sources and syndromes, frequency of data provision, attributes, etc.) was solicited. This section was completed and returned in advance of the interview. In section 2 of the semi-structured interview, information was collected on the timeliness, validity (measured by whether the system detected wave two of the pandemic) and operation of systems used most frequently from the web-survey (sentinel physician consults for influenza-like illness (ILI), school absenteeism, ED visits and/or febrile respiratory illness (FRI) screening data from ED’s, and telephone helplines such as Telehealth), while the uses of these systems during the pandemic to inform specific public health actions was explored in section 3. The guide for NSU’s consisted of a modified version of Section 3, asking questions about actions taken during the pandemic and the role of surveillance data in informing these decisions. Specific public health decisions of interest concerned the operation of influenza assessment centres and immunization clinics, closing schools, sending health information home from schools, providing recommendations to health care providers, and providing information to the public about recommendations and influenza activity levels.

Telephone interviews were conducted privately with each participant in their workplace by two trained research staff (AC and RS) between November 2010 and January 2011, with the exception of two interviews where the participants requested a joint interview with a colleague(s). Prior to commencing the interview, the research staff, who were epidemiologists involved in pandemic surveillance and research, described the study’s goals. Interviews were digitally recorded and transcribed verbatim by a professional transcriptionist. Accuracy of each transcribed interview was verified with the digital recording by the researchers and revisions made as necessary.

### Data management and analysis

Section 1 responses were copied to an Excel file for analysis, while transcripts from section 2 and 3 interviews were uploaded into NVivo 8® to assist with data organization, coding and analysis.

A coding scheme was developed based on the CDC framework [5] and the interview guide, and modified based on specific study objectives, study team discussions, feedback from the Advisory Committee and pilot testing. A deductive approach was used to assign the data to overarching themes or categories for analysis that reflected this scheme. Each interview was coded by one of the two researchers who also conducted the interviews. Consistency in coding between the two research staff was tested with four non-pilot interviews prior to independent coding, at which time discrepancies were resolved to facilitate consistency. One interview was coded by both researchers at coding completion to confirm coding consistency over time.

NVivo® was used to code data and construct queries. Within the overarching themes defined by the coding scheme, a combination of content and thematic analysis was used to analyze the interviews. Thematic analysis was performed to identify emerging themes or patterns using an inductive approach within and across the categories defined by the coding scheme. To aid in the recognition of these patterns, the constant comparison method was used to determine how different pieces of data were similar or different [16]. Content analysis was then performed to quantify the frequency of the emerging themes or sub-categories. The emerging themes were discussed amongst the research team, revised, and then presented to the study’s Advisory Committee for review, who deemed the themes credible based on their understanding of pandemic surveillance in the field.

### Ethics approval

Ethics approval was obtained from University of Toronto’s Office of Research Ethics. Interviews were conducted only after informed, written consent was obtained from the study participants.

## Results

A description of study participants and their syndromic surveillance systems is first presented to provide contextual information on the views being represented and the syndromic data participants refer to in their interviews. Following this, three key themes describing the value of syndromic data for decision making are presented: beneficial impacts; areas of limited impact and the role of other influences; and limitations of syndromic data.

### Study participants

**Figure 1:**
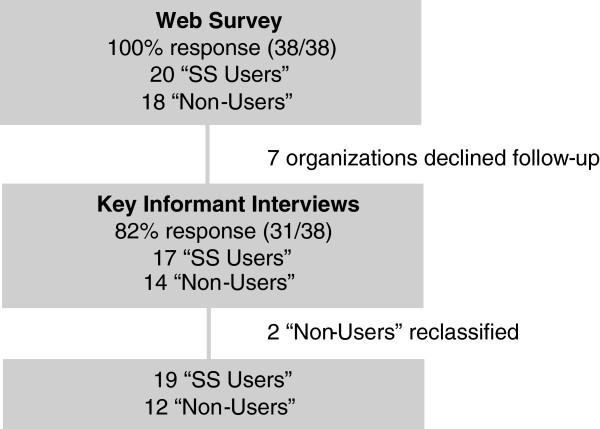
Flow chart of study participants.

Overall, 31/33 (94%) of invited organizations agreed to participate for an overall response rate of 82% (31/38). The seven organizations who declined participation were local public health departments of smaller and medium size (2/9 departments serving a population size of <125,000 and 5/13 departments serving a population size of 125,000-400,000 declined). According to the web-survey, four of the non-responders were classified as NSUs and three as SSUs (Figure 1). For the 31 participating organizations, 19 met the definition for SSUs (Figure 1). Among the 56 interviews conducted (involving 58 individuals), 34 interviews were from SSUs and 22 from NSUs. In five organizations, only one interview was completed; and in one organization, three interviews were conducted to account for the distribution of responsibilities across individuals during the pandemic. In addition to epidemiologists and medical officers of health, who comprised 39% and 29% of participants, other participants included managers (19%), directors (7%), and nurses (5%).

### Syndromic surveillance system descriptions

In total, 31 syndromic surveillance systems were in operation at the time of interview among the 19 syndromic surveillance users; 26 of which were in operation during the pandemic (Table 2). In Ontario, school absenteeism and ED data were the most frequently used data sources for syndromic surveillance.

**Table 2:** Characteristics of Ontario syndromic surveillance systems, overall (N = 26) and by data source

**System characteristic**	**ED**^**a**^		**SA**^**a**^		**Other**^**a**^		**Overall**	
	**n**	**%**	**n**	**%**	**n**	**%**	**Total**	**%**
Operation Length								
*1 year or less*	2	22	4	40	1	14	7	27
*2-4 years*	3	33	5	50	1	14	9	35
*5 years or more*	4	44	1	10	5	71	10	38
Syndrome Based	9	100	4	40	6	86	19	73
Frequency of Data Provision								
*Real-time*	4	44	0	0	0	0	4	15
*Daily*	3	33	6	60	3	43	12	46
*Weekly*	2	22	4	40	4	57	10	38
Mechanism of Transfer^b^								
*Automated*	5	56	3	30	0	0	8	36
*Manual*	2	22	6	60	2	67	10	45
*Both components*	2	22	1	10	1	33	4	18
Frequency of Monitoring^c^								
*More than daily*	2	25	0	0	0	0	2	9
*Daily*	4	50	6	60	2	50	12	55
*Weekly*	1	13	4	40	2	50	7	32
*Other*	1	13	0	0	0	0	1	5
Threshold or Algorithm Used in Analysis	6	67	9	90	2	29	17	65
Response Protocol^c^	2	25	5	50	1	25	8	36

^a^*ED*, emergency department visits (n = 9); SA, school absenteeism (n = 10), and other includes daycare absenteeism (1), OTC pharmacy sales (1), sentinel ILI consultations (3), Telehealth (1) and workplace absenteeism (1).

^b^4 missing responses for Other systems. ^c^1 missing response for ED systems and 3 missing responses for Other systems.

With the exception of two non-custom syndromic surveillance systems, all systems were created independently by each local health department. As a result, there was substantial variation between systems, including syndrome definition, frequency and method of data transfer, and methods for aberrant event detection. Despite this, the process of investigating alerts was similar across systems. Epidemiologists would notify an internal infectious disease team, analyze the data to determine if there was clustering in age/grade or geography, triangulate syndromic data with other surveillance data and if warranted, call hospitals or schools to try to elucidate a potential cause for the increase.

### Role of syndromic data in informing public health actions

A summary of the role of syndromic data and other factors in supporting specific public health decisions and actions during the pandemic is shown in Table 3.

**Table 3:** **Role of syndromic surveillance and other factors in supporting public health decisions**^**a**^

**Public health decision**	**Syndromic data**	**Other factors supporting decision-making for SSUs**	**Factors supporting decision-making for NSUs**
**Useful for:**			
**Operation of influenza assessment centres,** e.g. *opening/closing, location*	ED data particularly used to support opening and in some instances, the location	Consultations with health care providers about pressure on the health care system	Consultations with health care providers about pressure on the health care system
		Assessment centre activity	Hospital decision
**Recommendations/communications to the public**	Overall, provided picture of virus activity in the community and burden on the health care system to inform the timing and content of communications	Inclusion of standard infection prevention and control recommendations	Inclusion of standard infection prevention and control recommendations
		Releasing communications regularly was made part of regular practice	Guidance from the MOHLTC and provincial teleconferences
	Overall, provided credibility in knowing the situation to help support and reinforce messages	To be proactive	Significant event, e.g. the first lab confirmed case or death
		Laboratory data were used to provide updates about community activity levels	Releasing communications regularly was made part of regular and collaborative practice with media
			Response to media requests
**Use of surveillance bulletins**	Overall, used to communicate and provide updates on virus activity levels in the community internally and externally	Providing/updating bulletins was made part of regular practice.	Providing/updating bulletins was made part of regular practice.
		Laboratory data were included	Laboratory data were included
**Minimal impact on:**			
**Operation of immunization clinics**, e.g. *opening/closing, location*	As a reflection of community activity, all data generally reinforced urgency of clinics and supported timing of closures	Vaccine supply	Vaccine supply
		Demand for vaccine	MOHLTC guidance
		MOHLTC guidance	Experiences with previous seasonal influenza campaigns
		Geographic distribution of population; physical adequacy of space to accommodate equipment, car parking and line-ups	Geographic distribution of population; physical adequacy of space to accommodate equipment, car parking and line-ups
**Closing schools**	Overall, data showed community-wide spread and thus, would not be helpful at preventing transmission	Understanding of the potential usefulness based on the research literature and societal impact if closed.	Understanding of the potential usefulness based on the research literature and societal impact if closed.
	For some local health departments, school absenteeism data did not suggest need to close.	MOHLTC guidance	MOHLTC guidance
**Sending information letters home with school children**	School absenteeism data identified schools for targeted communication about infection prevention and control measures	First lab confirmed case or death of a child	Vaccine availability for school-aged children
		Start of the school year	First lab confirmed case or death of a child
		New information available from the MOHLTC	New information available from the MOHLTC
			Requests by schools or school boards
**Recommendations/messages to health care providers**	Overall, data was used to update stakeholders regularly about H1N1 activity in the community and support communications with health care partners	Guidance from the MOHLTC and provincial teleconferences	Guidance from the MOHLTC and provincial teleconferences
			To maintain regular communications with health care partners

^a^MOHLTC, Ontario Ministry of Health and Long-Term Care.

#### Beneficial impacts

Related to beneficial impacts, participants in 16/29 (55%) of the SSU interviews reported that ED data were useful for informing the need for opening influenza assessment centres. Total daily ED visit counts or visits for ILI symptoms were used as indicators of the burden on the health care system and as signals for opening centres. Decreased demands in the ED and at the centres were signals for their closure.

"“We looked back at ___ hospitals and calculated a rough baseline during a normal flu season for visit volume and then we chose a threshold that if we went over that, we would open the assessment centres. As soon as this occurred, we opened the assessment centres that same day because they were planned and ready to go.” (Participant 23, epidemiologist)"

In addition to ED data, several SSUs (8/29 or 28%) reported relying on stakeholder consultation to inform the need for and timing of influenza assessment centres, particularly in the establishment of thresholds or triggers for opening centres. Syndromic data were also used by the majority of SSUs (23/27 or 85%) for notifying the public and stakeholders about A(H1N1)pdm09 activity in the community and informing the timing and content of communications made through organizational websites, surveillance bulletins or through the media. This information was felt to add credibility and confidence which supported and reinforced public health messages. Data from syndromic surveillance were also frequently summarized (25/30 or 83%) in surveillance bulletins to update staff within the organization, external stakeholders and the public on community A(H1N1)pdm09 activity.

"“It (syndromic surveillance data) would have informed the information they put into that, those communication press releases. So again, that’s about the risk communication side of things… People and the media want to know what the impact is on the community and the syndromic surveillance stuff is actually quite good for defining that.” (Participant 56, associate medical officer of health)"

"“It was most useful to me in public communication and communication to partners… It gave I think a level of confidence to the media that we were on top of it as well as we could be…That’s immensely helpful to be able to project confidence.” (Participant 32, medical officer of health)"

#### Areas of limited impact and the role of other influences

Where syndromic data had limited impact on decision-making was in the operation of immunization clinics (with the exception of timing their closure) (14/30 or 47%), school closures (3/28 or 11%), sending information home with school children (although 16/26 or 62% indicated syndromic data supported communications with schools) and recommendations and messages made to health care providers (6/29 or 21%). Respondents stated that vaccine availability (17/30 or 57%) and central guidance from Ontario’s Ministry of Health and Long-Term Care (MOHTLC) (6/30 or 20%) were driving factors in immunization clinic operations. Central recommendations were also influential in deciding not to close schools (8/28 or 29%) and in messages to health care providers, particularly with respect to infection control practices (14/29 or 48%). In some cases, no action was taken despite higher levels of activity.

"“We opened them (immunization clinics) as soon as we had the supplies… the data that was coming out of our system wasn’t used to dictate where they were placed. They were opened in spots that we normally would have our flu clinics and then the extra ones were put in more remote jurisdictions to decrease travel time for those residents who were more geographically isolated.” (Participant 23, epidemiologist)"

"“Well it (closing schools) wasn’t recommended right, so they were just following the guidelines. I mean they had lots of absenteeism. Schools were quite hard hit. But there was no point in closing them…” (Participant 54, associate medical officer of health)"

Like SSUs, public health decisions by NSU’s regarding the operation of immunization clinics were primarily informed by vaccine availability (12/20 or 60%), while MOHTLC guidance informed school closures (9/20 or 45%) and recommendations and messages made to health care providers (14/21 or 67%). Stakeholder consultations were also valued for providing information about pressures on the health care system and the need to open influenza assessment centres (10/20 or 50%), and as a means to maintain regular communications with partners (8/21 or 38%).

"“We communicated with our community partners in health care and in school and education. This information was updated and relayed to us at these teleconferences, how they felt they were managing, whether they felt they were getting overwhelmed and that kind of thing. So it was basically directly from them telling us how they were managing.” (Participant 37, senior public health nurse)"

Traditional surveillance from laboratory data were used to monitor and provide updates on community A(H1N1)pdm09 activity (5/21 or 24%) while communications with the public, including the use of surveillance bulletins were made part of routine public health practice (8/21 or 38%).

#### Limitations of syndromic surveillance data

Some limitations of syndromic data identified by participants included its poor specificity or ability to determine if alerts were due to true events/disease, timeliness (data were not received early enough to inform decisions) and reliability (data were not received consistently from data providers or were incomplete), particularly of reporting by sentinel community practitioners of ILI consultations. With many systems relatively newly implemented, several SSUs also expressed not having baseline information or algorithms/response protocols for interpreting syndromic data and deciding when to initiate a public health response.

## Discussion

This study contributes to a small but growing body of literature aimed at documenting whether and how syndromic data is linked with public health action, and additionally describes capacities to use these data. In the context of the recent pandemic in Ontario, this study shows that data from syndromic surveillance systems had limited application in decision-making regarding selected public health actions. Many of these decisions were instead driven by other logistical and contextual factors (e.g. vaccine availability and MOHTLC recommendations). Other influences on decision-making for both SSUs and NSUs were traditional surveillance from laboratory data and communications with stakeholders.

Syndromic surveillance data were most frequently used for communications and messaging, both internally within an organization and externally with stakeholders, partners and the media. Specifically, syndromic data were used in surveillance bulletins to communicate A(H1N1)pdm09 activity, improve risk communication and support recommendations, and in the operation (e.g. opening, closing and placement) of influenza assessment centres. Additionally, syndromic data were valued for monitoring virus activity in the community and providing credibility and confidence to support decisions and recommendations. These uses reflect a reactive approach to the impact of A(H1N1)pdm09 activity on the health care system, rather than a proactive approach to early identification and action (e.g. advanced set up of control systems such as assessment centres before health care systems were pressured).

Few studies have examined the public health actions taken in response to syndromic data. In the US, case studies have found that even when received in a timely manner, syndromic surveillance did not have an influence on public health actions for seasonal influenza [9]. In contrast, data from England and Wales’ national telephone health advice helpline, NHS Direct, have been used to track seasonal influenza and to communicate risk of adverse events and provide reassurance to the public following a fuel explosion [17,18].

US studies have also reported the use of syndromic data for monitoring of disease activity and communicating surveillance findings to stakeholders and the community [9,14,19]. Uncertainty about the ability of syndromic surveillance to detect outbreaks has led to its greater utility for situational awareness regardless of the method of detection, particularly for monitoring influenza activity and its public health impact [19]. The value of syndromic surveillance for monitoring disease activity in the US is reflected by the finding that 98% (40/41) of respondents (from state, territorial and large, local jurisdictions) indicated that they planned to use syndromic surveillance to monitor the impact of pandemic influenza [19].

In this study population, syndromic surveillance was most used for monitoring A(H1N1)pdm09 activity and its impact on the health care system, as well as supporting communications and messaging, rather than use for its intended purpose of early outbreak detection. SSUs valued the information about community disease activity that syndromic data provided and the reassurance and confidence it provided to decision-makers. However, if not linked to action or contributing to the prevention and control of adverse health related events, the utility and benefits of syndromic surveillance are unclear [5].

Given the use of syndromic surveillance to support and reinforce public health messages, this study raises questions about the value of syndromic surveillance as an adjunct to traditional influenza surveillance systems. It is unclear how many surveillance systems are needed if these systems are most beneficial for situational awareness and providing supportive evidence to increase credibility and confidence in decisions previously made. Further familiarity with syndromic surveillance systems and development of algorithms for generating and responding to alerts may improve its utility for decision-making. Even with such enhancements, the utility of syndromic surveillance data may continue to be constrained if other systemic barriers are not addressed. Determining how and under which conditions the utility of syndromic surveillance can be maximized requires further study.

The strengths of this study include good representation (82%) from decision-makers and data users across Ontario’s public health departments. The use of semi-structured interviews gave participants flexibility in their responses and allowed us to obtain a richer understanding of system experiences. Having decision end points and outcomes provided objectivity to evaluating the usefulness of syndromic data in supporting public health actions during the pandemic.

One important limitation of this study’s findings is the high level of variation we noted in the operation of syndromic surveillance systems. With 27% of systems in operation for less than two years, many users indicated a lack of experience or ability to use historical trends to define triggers for action, which likely contributed to its limited use for decision-making. These findings are similar to a study of eight US states where only 48% of SSUs had written response protocols, and where the lack of a systematic process for designing protocols and few available information resources for response were identified [14]. For systems in place for longer than two years, however, the reasons for not having triggers are unknown. Additionally, as this study asked about use of syndromic data for specific decision end points, whether syndromic surveillance had any additional indirect impacts on decision-making more generally (beyond providing reassurance and confidence) remains unanswered.

As respondents were limited to those from local, provincial and federal public health agencies, we also have not captured how syndromic data were used by data providers (e.g. hospitals, schools). Another study limitation includes the potential for recall bias as interviews were conducted almost a year after the pandemic peak. Although participants were assured of confidentiality, other possible biases include providing responses that are likely to be viewed more favourably, attract further funding, or as socially desirable, especially when much time and effort was invested in collecting and analyzing syndromic data.

## Conclusions

Syndromic surveillance systems had limited application in decision-making for many of the public health actions evaluated in this study. Despite this, syndromic data were valued for monitoring local virus activity which provided credibility and confidence to support public communications and recommendations. Developing an understanding of how syndromic data is utilized during public health events provides valuable evidence to support future investments in public health surveillance, especially when resources are limited and choices need to be made of where to invest. Further study is required to determine whether improvements to systems, such as automating and improving the frequency of data provision or developing and applying algorithms to generate and respond to alerts, can improve the utility of syndromic data for decision-making.

## Competing interests

No potential conflicts of interest (financial or otherwise) are declared by the authors.

## Authors’ contribution

AC participated in the study’s coordination, performed the interviews and analysis, and drafted the manuscript. RS conceived of the study, participated in its design, performed the interviews and analysis, and drafted the manuscript. IJ conceived of the study, participated in its design, assisted in the interpretation of the data and drafted the manuscript. DW, NSC, LCR, IG, and RFD conceived of the study, participated in its design and assisted in the interpretation of the data. DS, JG, and AW participated in the study design and assisted in the interpretation of the data. All authors read and approved the final manuscript.

## Pre-publication history

The pre-publication history for this paper can be accessed here:

http://www.biomedcentral.com/1471-2458/12/929/prepub

## Supplementary Material

Additional file 1 **Interview guide for syndromic surveillance users (SSUs) and non-syndromic surveillance users (NSUs).** This file outlines the order and type of questions asked of study participants. Click here for file
