# Supplementary material for: The use of syndromic surveillance for decision-making during the H1N1 pandemic: A qualitative study
Source: BMC Public Health. 2012 Oct 30;12:929. doi: 10.1186/1471-2458-12-929 (PMC3539916; doi:10.1186/1471-2458-12-929)
Supplement: Additional file 1 — Interview guide for syndromic surveillance users (SSUs) and non-syndromic surveillance users (NSUs). This file outlines the order and type of questions asked of study participants. [file 1471-2458-12-929-S1.pdf]

Additional File 1. Interview guide for syndromic surveillance users (SSUs) and non-syndromic surveillance users (NSUs).

Interview Guide for Phase 3 (SSUs)

Please complete Section 1 of the Interview Guide and send to [redacted]*@oahpp.ca one week prior to the interview. Note that the name(s) of the system(s) are pre-populated according to the information you provided in the web survey.*

Section 1: Description of Syndromic Surveillance Systems

1. How many syndromic surveillance systems does your health unit monitor?

   Enter Number |__|__| *(enter number, enter zero if none, blank is missing, or DK if "don't know").*

2. How many years have each of the systems been in place/operational?

Name of system	*Number of years in operation*	*Comments (recent changes)*

3. What is the major objective of each system?

Name of system	*Purpose*

4. What are the typical uses of the system?

Name of system	*Typical use*
Example: *Hospital Emergency Reporting system*	*Used to …*

5. What are the primary data sources for each system? And, what is the population coverage?

Name of system	*Data source*	*Population being covered*
Example: *Hospital Emergency Reporting system*	*Primary complaints of patients presenting at the emergency departments*	*Population of 40,000 persons living near the hospital*

6. How frequently is data provided or refreshed to you as a health unit?

Name of system	*Description of data/report provided*	*Frequency of data provision*
Example: *Hospital Emergency Reporting system*	*Summary reports of numbers of cases by symptom category analysed by CUSUM*	*Once per week*

7. What syndromes are monitored?

Name of system	*List of syndromes*
Example: *Hospital Emergency Reporting system*	• Fever and Respiratory Illness<br>• *Enteric illness, etc*

8. Were there any system outages or downtimes for servicing during periods of need through the pandemic?

Name of system	*Downtime or outage?*			*If yes, please describe:*
	Yes	No	Not sure	
Example: *Hospital Emergency Reporting system*	☐	☐	☐	
	☐	☐	☐	

9. Does the system regularly receive IT support, e.g. for an emergency?

Name of system	*System support?*			*If yes, please describe:*
	Yes	No	Not sure	
Example: *Hospital Emergency Reporting system*	☐	☐	☐	
	☐	☐	☐	

10. Were upgrades done on the system during the pandemic?

Name of system	*Upgrades done?*			*If yes, please describe:*
	Yes	No	Not sure	
Example: *Hospital Emergency Reporting system*	☐	☐	☐	
	☐	☐	☐	

11. How flexible is the system:
    a. to adjust syndrome classification criteria,
    b. to add additional data sources,
    c. to adjust detection thresholds, and
    d. to produce reports?

Name of system	*Ability to adjust syndrome classification criteria*	*Ability to add additional data sources*	*Ability to adjust detection thresholds*	*Ability to produce reports*
	[ ] Very flexible<br>[ ] Acceptable<br>[ ] Not very flexible<br>Comments:	[ ] Very flexible<br>[ ] Acceptable<br>[ ] Not very flexible<br>Comments:	[ ] Very flexible<br>[ ] Acceptable<br>[ ] Not very flexible<br>Comments:	[ ] Very flexible<br>[ ] Acceptable<br>[ ] Not very flexible<br>Comments:

12. Please describe the number of staff that monitor the syndromic surveillance system and include the total hours spent by all staff in monitoring systems and investigating alerts (not including full-blown epidemiological investigation).

Name of system	*Number of staff involved*	*Estimated total hours per week to monitor systems (A)*	*Estimated total hours per week to investigate alerts (B)*	*Estimated total hours per week (A+B)*

13. What are the total non-personnel costs to maintain the system (include start-up and ongoing costs)?

Name of system	*Fixed costs for hardware (desks computers, etc.) (A)*	*Fixed costs for software (computer programming) (B)*	*Estimated overall costs (A+B)*

14. In your opinion, what are the major strengths and limitations of the system?

Name of system	*Strengths*	*Limitations*
	e.g. received more detailed information than provided by traditional systems; enhanced communication with health care providers/schools, etc.	*e.g. system's specificity is low; false alerts, etc.*

Section 2: How the Syndromic Surveillance Data/Systems were used During H1N1

Please note that Sections 2 and 3 of the Interview Guide will be asked during the telephone interview.

15. Please answer the following questions for each data source/system listed below. Only applicable data sources/systems you identified in the web survey as used during the H1N1 pandemic are listed and will be asked during the interview.

**Questions**	**Data Source/System**<br>***Data source*** is defined as a collection of raw data whether a pile of papers or compilation of digital data.<br>***System*** is a pre-defined set of processes or steps through which information is generated, managed, analyzed and disseminated. It involves pre-determined steps or actions.				
	Sentinel physician ILI consultations (FluWatch and/or similar local systems)	School absenteeism	Emergency department visits (i.e. number of visits)	Emergency department screening data (i.e. reason for visit)	Organization telephone information line (i.e. local public health information line)
a. Using the definition given in the table, what do you think do you have – a system or a data source?					
b. Was Wave 2 of the pandemic detected through your data source/system?					
c. How timely was detection relative to the lab data?					

Questions	Data Source/System<br>***Data source*** is defined as a collection of raw data whether a pile of papers or compilation of digital data.<br>***System*** is a pre-defined set of processes or steps through which information is generated, managed, analyzed and disseminated. It involves pre-determined steps or actions.				
	Sentinel physician ILI consultations (FluWatch and/or similar local systems)	School absenteeism	Emergency department visits (i.e. number of visits)	Emergency department screening data (i.e. reason for visit)	Organization telephone information line (i.e. local public health information line)
d. How was your syndromic surveillance data source/system used at your health unit during the pandemic?<br><br>i.e. if used for outbreak detection during the pandemic, was it used for detecting the beginning of Wave 2 or for tracking the entire duration of the outbreak?					
e. Who was responsible for monitoring the data source/system?					

Questions	Data Source/System<br>***Data source*** is defined as a collection of raw data whether a pile of papers or compilation of digital data.<br>***System*** is a pre-defined set of processes or steps through which information is generated, managed, analyzed and disseminated. It involves pre-determined steps or actions.				
	Sentinel physician ILI consultations (FluWatch and/or similar local systems)	School absenteeism	Emergency department visits (i.e. number of visits)	Emergency department screening data (i.e. reason for visit)	Organization telephone information line (i.e. local public health information line)
f. How often were the data source/systems monitored?					
g. Describe the process of monitoring the data and specify timelines:<br><br>(see bulleted points below)					
• What detection algorithms were used/how were alerts generated?					
• How did you respond to alerts or alarming trends detected in the data? (drill down into system data, review line list, check for false positives, call hospital to inquire, request lab results, etc)?					

Questions	Data Source/System<br>***Data source*** is defined as a collection of raw data whether a pile of papers or compilation of digital data.<br>***System*** is a pre-defined set of processes or steps through which information is generated, managed, analyzed and disseminated. It involves pre-determined steps or actions.				
	Sentinel physician ILI consultations (FluWatch and/or similar local systems)	School absenteeism	Emergency department visits (i.e. number of visits)	Emergency department screening data (i.e. reason for visit)	Organization telephone information line (i.e. local public health information line)
• How were data generated by this source/system (including alerts) shared within your organization?					
• Were these data shared with others outside the health unit? And if so, to whom?					
• Were any standardized response protocols in place? Would you be willing to share this protocol? In general, were these followed during pH1N1? Why?					
h. Overall, how long does it take between getting the data and reporting the results?					

16. Regarding Telehealth:

    a. Did you receive any Telehealth alerts? If yes,

    b. How did you respond to these alerts?

    c. Was Telehealth data different from other data sources?”

17. a. How was information from the syndromic surveillance system triangulated or verified with other data sources or surveillance systems?

    b. What other data were most valuable in terms of verifying alerts or making comparisons?

    c. If there was disagreement among data sources, how was this handled?

18. In what ways did having access to multiple sources of surveillance information affect the decisions that you or your agency made?

Section 3: Usefulness of Syndromic Surveillance Systems during H1N1

19. Did your organization take any of the following actions during the pandemic, and was data collected by syndromic surveillance systems used to inform any of these actions?

If **Yes** → Please provide examples that describe how these data were used and for what purposes, explaining the role of each system used.
→ What were your criteria for activating these responses and were these criteria based on thresholds observed in the data?

If **No** → Please describe why data were not used (e.g. resource limitations, etc.).

Actions	*Was this action taken?*	*Was syndromic surveillance data used to make this decision?*	*Description of decision making process and criteria used*
Immunization clinics		[ ] Yes<br>[ ] No<br>[ ] Not sure	*e.g. school absenteeism data were used to increase or decrease the number of immunization clinics*
Flu assessment centres	[ ] Yes<br>[ ] No<br>[ ] Not sure	[ ] Yes<br>[ ] No<br>[ ] Not sure	*e.g. school absenteeism data were used to decide on when to open and/or close flu assessment centres*
Information letters sent home for schoolchildren	[ ] Yes<br>[ ] No<br>[ ] Not sure	[ ] Yes<br>[ ] No<br>[ ] Not sure	
School closure	[ ] Yes<br>[ ] No<br>[ ] Not sure	[ ] Yes<br>[ ] No<br>[ ] Not sure	

Recommendations to public	[ ] Yes<br>[ ] No<br>[ ] Not sure	[ ] Yes<br>[ ] No<br>[ ] Not sure	
Communication and Press Releases	[ ] Yes<br>[ ] No<br>[ ] Not sure	[ ] Yes<br>[ ] No<br>[ ] Not sure	
Recommendations to health care providers and/or institutions	[ ] Yes<br>[ ] No<br>[ ] Not sure	[ ] Yes<br>[ ] No<br>[ ] Not sure	
Release of surveillance bulletins	[ ] Yes<br>[ ] No<br>[ ] Not sure	[ ] Yes<br>[ ] No<br>[ ] Not sure	
Other: ___________________	[ ] Yes<br>[ ] No<br>[ ] Not sure	[ ] Yes<br>[ ] No<br>[ ] Not sure	

20. Which factors do you think facilitate successful utilization of syndromic surveillance data during the pandemic?

21. Which factors do you think limit the ability to use these data? *e.g. are there challenges specific to a system?*

22. What are the strengths and weaknesses of the systems in combination?

23. Overall, were the data able to support inferences on the progression of the pandemic, in terms of burden of illness and severity? (Yes or No)

24. It has been noted that syndromic surveillance data can provide reassurance to support decision making. On the other hand, some literature suggests that it can lead to confusion and impede in decision making process. From your perspective, do syndromic surveillance data provide ***reassurance*** or ***impede*** in making decisions during a pandemic?

The following questions focus on your overall experience in using syndromic data individually and in combination as well as your future plans.

25. You ranked _____________________as your first choice among all sources of surveillance information you would like to monitor, can you please explain why?

26. If you already have your first choice, would you still consider monitoring the other top three sources that you indicated in the web survey (_____________________, _____________________, _______________________)? Why? e.g. *What would be the additional benefits or disadvantages?*

27. Do you think it would be useful to have something monitored for you at the central/provincial level?

28. What is the difference in the surveillance experience now and before the syndromic surveillance systems were in place?

29. Based on your organization's experience during the pandemic, are you likely to expand or reduce your use of syndromic surveillance in the next few years?

Additional questions:

30. Would you be interested in receiving findings about the study?

31. Is there a particular mechanism or method you prefer when receiving information about the study? *e.g. report, in-person presentation, webinar*

32. Would it be alright to contact you in the future to clarify some of the information you provided us?

Interview Guide for Phase 3 (NSUs)

Section 3: Usefulness of Syndromic Surveillance Systems during H1N1

1. Did your organization take any of the following actions during the pandemic?

Action	*Taken or not*	*Description of decision making process (i.e. why was action taken or not taken?)*
Immunization clinics		*e.g. Please explain the decision making process regarding increasing or decreasing the number of immunization clinics open*
Flu assessment centres	[ ] Yes<br>[ ] No<br>[ ] Not sure	
Information letters sent home for schoolchildren	[ ] Yes<br>[ ] No	

	[ ] Not sure	
School closure	[ ] Yes<br>[ ] No<br>[ ] Not sure	
Recommendations to public	[ ] Yes<br>[ ] No<br>[ ] Not sure	
Communication and Press Releases	[ ] Yes<br>[ ] No<br>[ ] Not sure	
Recommendations to health care providers and/or institutions	[ ] Yes<br>[ ] No<br>[ ] Not sure	
Release of surveillance bulletins	[ ] Yes<br>[ ] No<br>[ ] Not sure	
Other: __________________	[ ] Yes<br>[ ] No<br>[ ] Not sure	

2. Regarding Telehealth:

   a. Did you receive any Telehealth alerts? If yes,

   b. How did you respond to these alerts?

   c. Were Telehealth data different from other data sources?"

3. a. What data were most valuable in terms of verifying alerts or making comparisons?

   b. How was information from local surveillance systems triangulated or verified with other information and directives?

   c. If there was disagreement among data sources, how was this handled?

4. Did you review syndromic surveillance data from other jurisdictions? If yes, what did you use that information for?

5. Thinking about the way you made decisions during H1N1, do you think there would have been an added benefit from having your own local syndromic data?

6. What factors (financial, staffing/resources, access to data, etc) limit your use of syndromic surveillance data?

7. From your perspective, will syndromic surveillance data provide ***reassurance*** or ***impede*** in making decisions during a pandemic?

8. You ranked __________________________ as your first choice among all sources of surveillance information you would like to monitor. Can you please explain why?

9. If you already monitor your first choice, would you still consider monitoring the other top three sources that you indicated in the web survey (__________________, ____________________, and _______________________)? Why? *What would be the additional benefits or disadvantages?*

10. Do you have plans to develop or adapt a syndromic surveillance system in your unit?

Additional questions:

11. Would you be interested in receiving findings about the study?

12. Is there a particular mechanism or method you prefer when receiving information about the study? *e.g. report, in-person presentation, webinar*

13. Would it be alright to contact you in the future to clarify some of the information you provided us?
